# Localization of Laplacian eigenvectors on random networks

**DOI:** 10.1038/s41598-017-01010-0

**Published:** 2017-04-25

**Authors:** Shigefumi Hata, Hiroya Nakao

**Affiliations:** 1 Department of Physics and Astronomy, Kagoshima University, Kagoshima, 890-0065 Japan; 2 Department of Systems and Control Engineering, Tokyo Institute of Technology, Tokyo, 152-8552 Japan

## Abstract

In large random networks, each eigenvector of the Laplacian matrix tends to localize on a subset of network nodes having similar numbers of edges, namely, the components of each Laplacian eigenvector take relatively large values only on a particular subset of nodes whose degrees are close. Although this localization property has significant consequences for dynamical processes on random networks, a clear theoretical explanation has not yet been established. Here we analyze the origin of localization of Laplacian eigenvectors on random networks by using a perturbation theory. We clarify how heterogeneity in the node degrees leads to the eigenvector localization and that there exists a clear degree-eigenvalue correspondence, that is, the characteristic degrees of the localized nodes essentially determine the eigenvalues. We show that this theory can account for the localization properties of Laplacian eigenvectors on several classes of random networks, and argue that this localization should occur generally in networks with degree heterogeneity.

## Introduction

Localization of eigenmodes is a well known phenomenon in many fields of science, with the Anderson localization in disordered systems providing a prominent example^1,2^. The localization properties of eigenvectors in random matrix models of disordered media have been studied intensively^3^. Applications of the random matrix theory to cross-correlation matrices of economic data have also been performed, and have revealed localized eigenvectors, which implies functional substructures in the data^4^.

In this study, we focus on the Laplacian matrices of random networks, which describes diffusion processes in various models of network-organized systems, such as random walks, epidemic spreading, information flow, coupled nonlinear oscillators, and activator-inhibitor systems^5–8^. In many cases, the properties of the Laplacian eigenvectors play decisive roles in the network dynamics. Similar to the Fourier eigenmodes of the ordinary Laplacian operator in spatially extended systems, the Laplacian eigenvectors provide natural “coordinates” for describing the dynamics on networks.

A remarkable property of the Laplacian eigenvectors on random networks is their localization with respect to the node degrees. Namely, the components of each eigenvector take relatively large values on a particular subset of nodes, while taking small values otherwise. Moreover, the localized nodes have *similar* degrees, i.e., numbers of edges, and this characteristic degree corresponds closely with the Laplacian eigenvalue (We say that a pair of nodes have “similar degrees” when the difference in their degrees is sufficiently smaller than the range of the entire degree distribution; see the Results section for a discussion on the similarity in node degrees). This localization property has significant consequences for the dynamics of network-organized systems. For example, in the pattern formation in network-organized reaction-diffusion systems, the Laplacian eigenvectors determine the critical modes at the onset of instability and often dominate the developed patterns in the nonlinear regime^9–11^. In coupled oscillators on networks, the Laplacian eigenvectors determine the synchronization dynamics of the oscillators^12–14^.

The localization of the Laplacian eigenvectors was first reported by McGraw and Menzinger in their numerical analysis of coupled phase oscillators on several classes of random networks^13^, and later utilized in the analysis of network-Turing patterns^9–11^. Localized Laplacian eigenvectors have also been investigated in a few specific classes of networks^15,16^. However, despite its apparent ubiquity and importance in dynamical processes on networks^7–14,17^, a clear theoretical explanation of the localization mechanism has so far been lacking. Therefore, the class of networks in which this localization can be observed remains unclear.

In this study, we propose a simple theoretical approach to analyze the origin of the localization of Laplacian eigenvectors for a general class of networks. Based on the perturbation analysis of the Laplacian matrix, we argue that the localization should generally occur in networks with degree heterogeneity. For illustration, we analyze localization properties of Laplacian eigenvectors in several classes of random networks.

## Results

### Laplacian matrix and its eigenvectors

We consider a network consisting of *N* nodes. The network topology is specified by a *N* × *N* adjacency matrix **A**, whose element ${A}_{ij}$ takes a value of 1 if there is an edge between nodes *i* and *j*, and 0 otherwise ($i,j=1,2,\cdots ,N$). We assume that the network is connected (a path exists between arbitrary nodes), and that the connection is non-directed, i.e., ${A}_{ij}={A}_{ji}$. In this study, we define the Laplacian matrix ${\bf{L}}=\{{L}_{ij}\}$ of the network as ${L}_{ij}={A}_{ij}-{k}_{i}{\delta }_{i,j}$, where ${k}_{i}={\sum }_{j=1}^{N}{A}_{ij}$ is the degree of the *i*th node, i.e., the number of edges, and ${\delta }_{i,j}$ is the Kronecker’s delta symbol^18,19^. As we will show, it is convenient to sort the node indices {*i*} in decreasing order of the degree *k*_*i*_, so that inequalities ${k}_{1}\ge {k}_{2}\ge \cdots \ge {k}_{N}$ hold. We denote the average degree of the network as $\langle k\rangle ={\sum }_{i=1}^{N}{k}_{i}/N$.

Diffusion processes on the network are described by the Laplacian matrix. Suppose that each network node is occupied by some substance *X*, and denote its concentration on node *i* as ${[X]}_{i}={x}_{i}$. The change in the concentration by the diffusive transportation of *X* is described as $d{x}_{i}/dt={\sum }_{j=1}^{N}{L}_{ij}{x}_{j}={\sum }_{j=1}^{N}{A}_{ij}({x}_{j}-{x}_{i})$, where the flux of the substance from node *j* to node *i* is proportional to the concentration difference *x*_*j*_ − *x*_*i*_ (Fick’s law).

The eigenvector ${\overrightarrow{\varphi }}^{(\alpha )}=({\varphi }_{1}^{(\alpha )},{\varphi }_{2}^{(\alpha )},\cdots ,{\varphi }_{N}^{(\alpha )})$ and the eigenvalue ${{\rm{\Lambda }}}^{(\alpha )}$ of the Laplacian matrix **L** satisfy the eigenvalue equation

1 $$\sum _{j=1}^{N}{L}_{ij}{\varphi }_{j}^{(\alpha )}={{\rm{\Lambda }}}^{(\alpha )}{\varphi }_{i}^{(\alpha )}\quad (i=1,...,N),$$

where $\alpha =\mathrm{1,}\,2,\cdots ,N$ is the index of the eigenvector. The eigenvectors can be orthonormalized as ${\sum }_{i=1}^{N}{\varphi }_{i}^{(\alpha )}{\varphi }_{i}^{(\beta )}={\delta }_{\alpha ,\beta }$ for $\alpha ,\beta =\mathrm{1,}\,2,\cdots ,N$, because **L** is a real symmetric matrix. The Laplacian matrix **L** is negative semidefinite, i.e., ${\sum }_{i,j}{x}_{i}{L}_{ij}{x}_{j}=-{\sum }_{i,j}{A}_{ij}{({x}_{i}-{x}_{j})}^{2}\le 0$ is satisfied for any vector $\overrightarrow{x}=({x}_{1},\cdots ,{x}_{N})$ ^6^. Therefore, all Laplacian eigenvalues are non-positive, and only one of them, which corresponds to the uniform eigenvector $(1,\cdots ,1)/\sqrt{N}$, takes 0 because the network is connected. The eigenvector indices {*α*} are also sorted in increasing order of the Laplacian eigenvalues so that ${{\rm{\Lambda }}}^{\mathrm{(1)}}\le {{\rm{\Lambda }}}^{\mathrm{(2)}}\le \cdots \le {{\rm{\Lambda }}}^{(N)}=0$ hold.

### Localization of Laplacian eigenvectors

**Figure 1:**
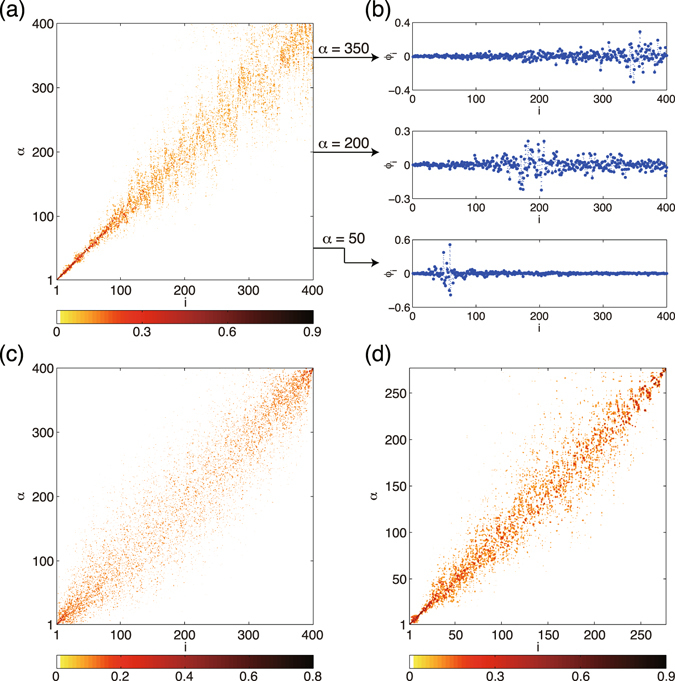
Laplacian eigenvectors. The absolute values of the vector components $|{\varphi }_{i}^{(\alpha )}|$ are shown for (**a**) Barabási-Albert (BA), (**c**) Erdös-Rényi (ER), and (**d**) *C. elegans* (CE) networks in contour plot. The network size and mean degree are fixed at $N=400$ and $\langle k\rangle =20$, respectively, for the BA and ER networks. The size of the CE network is $N=277$ and the mean degree is $\langle k\rangle \simeq 13.85$. For the BA network, three different eigenvectors corresponding to *α *= 50, 200, and 350 are shown in (**b**).

First, let us illustrate the Laplacian eigenvectors for several classes of random networks. In Fig. 1, all eigenvectors (except the uniform eigenvector ${\overrightarrow{\varphi }}^{(N)}=(1,\cdots ,1)/\sqrt{N}$ with ${{\rm{\Lambda }}}^{(N)}=0$, which has exceptional characteristics and is excluded from the analysis) of the scale-free network generated by the Barabási-Albert preferential attachment algorithm (BA)^20^, classical Erdös-Rényi random network (ER)^21^, and real neural network of *C. elegans* (CE)^22,23^ are displayed in the contour plot, where the horizontal axis is the node index and the vertical axis is the eigenvector index. We show the absolute value $|{\varphi }_{i}^{(\alpha )}|$ of the eigenvector components, because each component is statistically symmetric with respect to ${\varphi }_{i}^{(\alpha )}\iff -{\varphi }_{i}^{(\alpha )}$. For the BA network, three typical eigenvectors with different *α* are also shown for illustration. For the CE network, we focus only on the connectivity and symmetrize the original network, which consists of 277 neurons with directed connections, by defining the adjacency matrix as ${A}_{ij}={A}_{ji}=1$ if there is a edge between nodes *i* and *j*.

Remarkably, clear diagonal structures are observed in all figures. Because the nodes are sorted by their degrees, this means that, in each eigenvector, only the nodes sharing similar degrees take large vector components, while other nodes have very small components. Indeed, for each eigenvector shown in Fig. 1, the mean difference in the degrees of the localized nodes where $|{\varphi }_{i}^{(\alpha )}| > 0.1$ is 1.33 for the BA network, 2.01 for the ER network, or 2.10 for the CE network. Each of these numbers is much smaller than the entire range of the degree distribution in each network, *k*_1_ − *k*_*N*_, which is 106 for the BA, 25 for the ER, or 75 for the CE.

**Figure 2:**
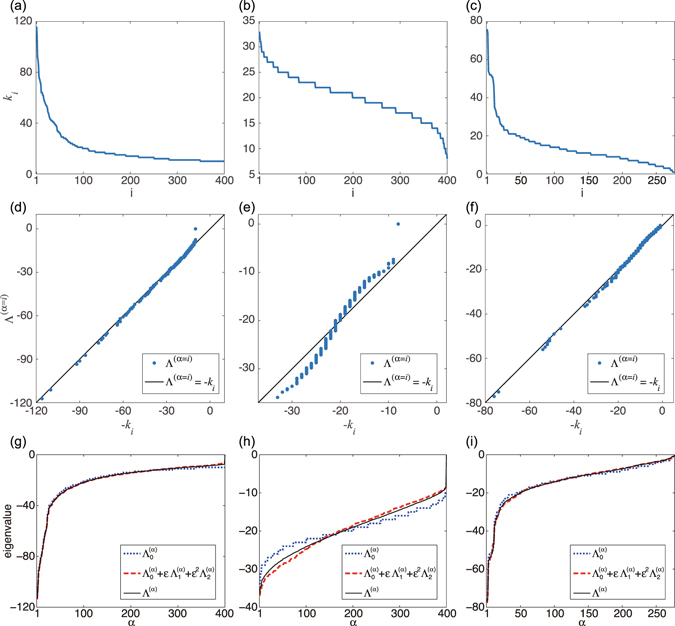
(**a–c**) Node degrees vs. node indices for the (**a**) BA, (**b**) ER, and (**c**) CE networks. (**d–f**) Scatter plots of degree-eigenvalue pairs, $({k}_{i},{{\rm{\Lambda }}}^{(\alpha =i)})$ ($i=1,\mathrm{...},N$), for (**d**) BA, (**e**) ER, and (**c**) CE networks. (**g–i**) Eigenvalues of the (**g**) BA, (**h**) ER, and (**i**) CE networks. Approximate eigenvalues obtained by the zeroth- and second-order perturbation theory are compared with the true eigenvalues obtained by direct numerical analysis.

Moreover, the visible diagonal structures indicate that the characteristic degree of each localized subset linearly correlates with the eigenvalue index, which is also sorted by their eigenvalues. Thus, clear degree-eigenvalue correlation exists in the Laplacian eigenvectors (See Fig. 2(c–f)). It is also notable that the patterns of the localization are qualitatively different among the networks. In the BA network [Fig. 1(a)], the localization is stronger near the hubs, i.e., the nodes with large degrees (e.g., *α* = 50 in panel (b)), while comparatively weak at the peripheries, i.e., the nodes with small degrees (e.g., *α* = 350 in panel (b)). In contrast to the BA network, in the ER [Fig. 1(c)] and CE networks [Fig. 1(d)], the localization is stronger both at hubs and peripheries, and weaker at the intermediate nodes. In McGraw and Menzinger^13^, the level of localization has been quantified by using the inverse participation ratio, i.e., ${\sum }_{i}{({\varphi }_{i}^{(\alpha )})}^{4}/{\{{\sum }_{i}{({\varphi }_{i}^{(\alpha )})}^{2}\}}^{2}$, a standard quantity used in the analysis of Anderson localization.

### Perturbation analysis of the Laplacian matrix

To analyze the origin of this intriguing localization property, we apply the perturbation theory^24,25^ to the Laplacian matrix. A similar perturbation approach was used by Kim and Motter to analyze the Laplacian *eigenvalues* of scale-free networks^26^.

In the present problem, the Laplacian matrix **L** has two types of elements of distinct orders. The diagnoal elements $-{k}_{i}{\delta }_{i,i}$ are order 〈*k*〉, while the non-diagional elements, which take 0 or 1, are ${\mathcal{O}}\mathrm{(1)}$. By introducing an expansion parameter $\varepsilon ={\langle k\rangle }^{-1}$, we can rewrite the Laplacian matrix as **L **= **L**_0_ + *ε* **L**_1_, whose elements ${L}_{\mathrm{0,}ij}=-{k}_{i}{\delta }_{i,j}$ and ${L}_{\mathrm{1,}ij}=\langle k\rangle {A}_{ij}$ are of the same order, ${\mathcal{O}}(\langle k\rangle )$. When the network is sufficiently dense, i.e., $\langle k\rangle \gg 1$, the expansion parameter *ε* is small, and it is expected that the perturbation theory yields reasonable approximation of the Laplacian eigenvectors.

For convenience, we employ the bra-ket notation to denote the Laplacian eigenvector, i.e., ${\overrightarrow{\varphi }}^{(\alpha )}=|\alpha \rangle$, and drop the summation symbol as ${\sum }_{j=1}^{N}{L}_{ij}{\varphi }_{j}^{(\alpha )}=L|\alpha \rangle$. Expanding the Laplacian eigenvectors |*α*〉 and eigenvalues ${{\rm{\Lambda }}}^{(\alpha )}$ in series of *ε* as $|\alpha \rangle ={|\alpha \rangle }_{0}+\varepsilon {|\alpha \rangle }_{1}+{\varepsilon }^{2}{|\alpha \rangle }_{2}+\cdots$ and ${{\rm{\Lambda }}}^{(\alpha )}={{\rm{\Lambda }}}_{0}^{(\alpha )}+\varepsilon {{\rm{\Lambda }}}_{1}^{(\alpha )}+{\varepsilon }^{2}{{\rm{\Lambda }}}_{2}^{(\alpha )}+\cdots$, and substituting into the eigenvalue equation (1), the following set of equations is obtained up to ${\mathcal{O}}({\varepsilon }^{2})$:

2 $$({L}_{0}-{{\rm{\Lambda }}}_{0}^{(\alpha )}){|\alpha \rangle }_{0}=\mathrm{0,}$$

3 $$({L}_{0}-{{\rm{\Lambda }}}_{0}^{(\alpha )}){|\alpha \rangle }_{1}=-({L}_{1}-{{\rm{\Lambda }}}_{1}^{(\alpha )}){|\alpha \rangle }_{0},$$

4 $$({L}_{0}-{{\rm{\Lambda }}}_{0}^{(\alpha )}){|\alpha \rangle }_{2}=-({L}_{1}-{{\rm{\Lambda }}}_{1}^{(\alpha )}){|\alpha \rangle }_{1}+{{\rm{\Lambda }}}_{2}^{(\alpha )}{|\alpha \rangle }_{0}\mathrm{.}$$

Let us first consider the unperturbed system (2). One can easily find that the eigenvectors ${|\alpha \rangle }_{0}$ and eigenvalues ${{\rm{\Lambda }}}_{0}^{(\alpha )}$ are given exactly as

5 $${|\alpha \rangle }_{0}=(0,\,\cdots ,0,\,\mathop{\mathop{1}\limits^{\vee }}\limits^{\alpha },0,\,\cdots ,0)\quad {\rm{a}}{\rm{n}}{\rm{d}}\quad {{\rm{\Lambda }}}_{0}^{(\alpha )}=-{k}_{\alpha }$$

for $\alpha =\mathrm{1,}\,\mathrm{...,}\,N$. Each eigenvector is characterized by a single non-vanishing element at the network node $i=\alpha$, and the corresponding eigenvalue ${{\rm{\Lambda }}}_{0}^{(\alpha )}$ is simply equal to the negative of the characteristic node degree ${k}_{\alpha }$. Thus, strictly localized eigenvectors are obtained at the zeroth-order, where the network topology is completely ignored and the non-diagonal elements are assumed to be vanishingly small. Note that ${{\bf{L}}}_{0}$ is no longer a Laplacian matrix because its row sums do not vanish. Also, unlike ${{\rm{\Lambda }}}^{(N)}$, which always vanishes, the zeroth-order eigenvalue ${{\rm{\Lambda }}}_{0}^{(N)}$ is not zero but equal to $-{k}_{N}$, i.e., the smallest degree of the network.

In order to analyze the localization property of the Laplacian eigenvectors, we should consider the higher-order perturbation terms and, in particular, the fact that networks generally possess multiple nodes with the same degrees. From the zeroth-order solution (5), this indicates that the zeroth-order eigenvectors are degenerate. Therefore, we need to employ the degenerate perturbation theory^24,25^. From Eqs (2–4), we can compute the approximate eigenvectors and eigenvalues by the first- and second-order degenerate perturbation theory, respectively. The complete derivation of the perturbation corrections for a general class of degenerate systems has been reported, e.g., in ref.^25^. See Methods and Supplementary Information for details. Accuracy of the perturbation approximation is also discussed in the Methods.

### Approximate eigenvectors

**Figure 3:**
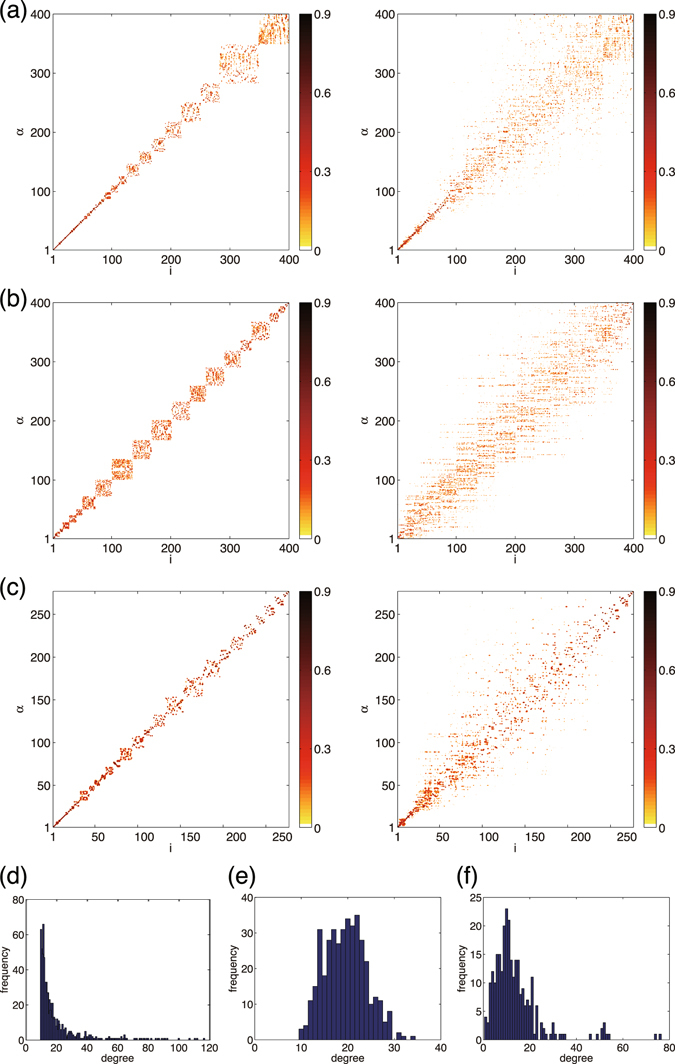
(**a–c**) Approximations of the Laplacian eigenvectors by the degenerate perturbation theory and (**d–f**) histograms of the degrees for (**a,d**) BA, (**b,e**) ER, and (**c,f**) CE networks. In panels (a–c), results of the zeroth-order (left) and the first-order (right) perturbations are shown.

**Figure 4:**
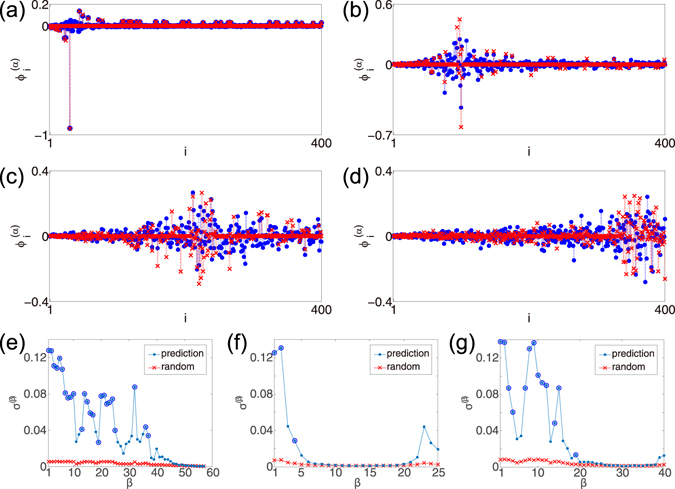
(**a–d**) Comparison of the approximate (red crosses) and true (blue dots) eigenvectors of the BA network for (**a**) $\alpha =30$, (**b**) $\alpha =95$, (**c**) $\alpha =231$, and (**d**) $\alpha =366$. Degeneration is not resolved at the first-order perturbation for eigenvectors shown in panels (c) and (d). (**e–g**) Correlation coefficient $\sigma$ between the true and approximate eigenvectors (blue dots) for the (**e**) BA, (**f**) ER, and (**g**) CE networks. Non-degenerate eigenmodes are indicated by circles. In each panel, correlation coefficient between the true and random eigenvectors are also plotted (red crosses).

**Figure 5:**
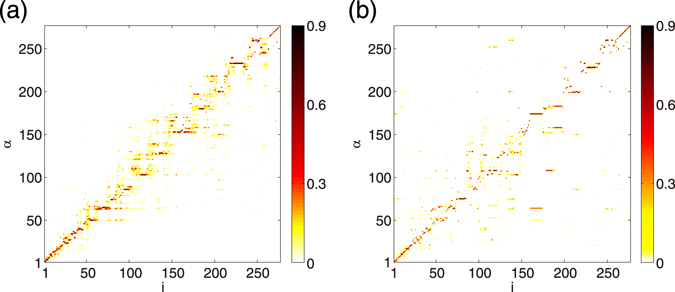
Laplacian eigenvectors of a real neural network of *C. elegans*. The magnitude of each vector component is shown for (**a**) true eigenvectors obtained by direct numerical diagonalization and (**b**) approximate eigenvectors obtained by the perturbation approximation.

**Figure 6:**
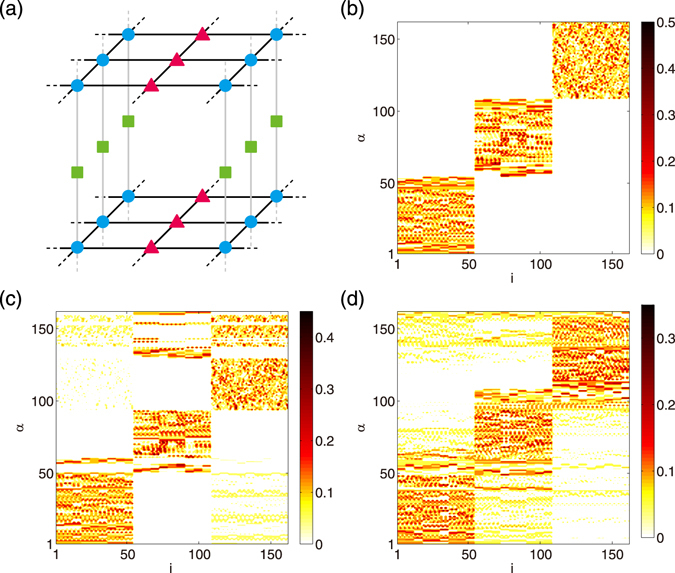
Laplacian eigenvectors of a lattice network with heterogeneous degrees. (**a**) Lattice network consisting of nodes of degree $k=6$ (blue circles), $k=4$ (red triangles), and $k=2$ (green squares). The network size is $N=162$. Periodic boundary conditions are employed. (**b,c**) Approximations of the Laplacian eigenvectors with the (**b**) zeroth- and (**c**) first-order perturbations. (**d**) True Laplacian eigenvectors obtained by direct numerical calculation.

We now apply the perturbation theory to the networks used in Fig. 1, and demonstrate that it can predict the localization properties of random networks. The results are shown in Figs 2–6.

Figure 2(a–c) show the node degrees of the BA, ER, and CE networks as functions of the node index. Because the node indices are sorted in decreasing order of degrees, the curves monotonically decreases with the node index. Figure 2(d–f) show scatter plots of degree-eigenvalue pairs, $({k}_{i},{{\rm{\Lambda }}}^{(\alpha =i)})$ $(i=1,\mathrm{...},N)$, for the three networks. We can observe that the data points approximately lie along the diagonal line in each figure, implying that the eigenvalues and node degrees are closely correlated in these networks. Such correlation between eigenvalues and degrees has also been reported in a preceding study^27^.

Now, Fig. 2(g–i) show the zeroth- and second-order approximations of the Laplacian eigenvalues. We can see that the zeroth-order result already provides a good approximation to the true Laplacian eigenvalues for the BA and CE networks. For the ER network, the zeroth-order eigenvalues somewhat deviate from the true eigenvalues, but higher-order approximation gives closer values. Thus, the perturbation theory accounts for the Laplacian eigenvalues of these networks, which indicates that they are essentially determined by the node degrees.

Figure 3 displays the approximated eigenvectors as a function of the eigenvector index *α* and the node index *i*, similarly to Fig. 1. As can be seen, the first-order predictions are in good qualitative agreement with the true Laplacian eigenvectors shown in Fig. 1. The diagonal structures indicating localization of eigenvectors are well reproduced for all networks. Moreover, the different patterns of localization among the networks are correctly reproduced. That is, the localization is stronger at hubs and weaker at peripheries in the BA network, while it is strong both at hubs and peripheries and weak at the intermediate nodes in the ER and CE networks.

The slightly broadened localization patterns along the diagonal line can be interpreted as follows. If some nodes in the network share the same degree, the corresponding zeroth-order eigenvectors are degenerate. The eigenvectors in the degenerate subspace are mutually mixed, yielding block-diagonal structures of various sizes in the contour plot. The size of each block-diagonal component is equal to the number of the degenerate eigenvectors, i.e., the number of nodes sharing the same degree. Therefore, the degree distribution of the network determines the pattern of localization.

Indeed, in the BA network, the degrees obey a scale-free distribution, where only a small number of nodes have large degrees (hubs) and the majority of the nodes have small degrees (peripheries) [Fig. 3(d)]. Correspondingly, the degeneracy of the degrees, i.e., the zeroth-order eigenvalues, is small for the hubs and large for the peripheries. Therefore, the localization is stronger at the hubs than that at the peripheries because less eigenvectors are involved.

In contrast, the ER network has a binomial distribution of the degrees [Fig. 3(e)]. The majority of the nodes belong to the intermediate degrees, and the hubs and peripheries are composed of relatively small numbers of nodes. This leads to stronger localization at both hubs and peripheries, and weaker localization at intermediate nodes, in contrast to the BA case. Similarly, in the CE network, the degrees obey a binomial-like distribution [Fig. 3(f)], so the localization pattern is also similar to that of the ER network.

In Fig. 4, the approximate eigenvectors of the BA network are compared with the true eigenvectors for several values of *α*. The eigenvectors shown in Figs (a) and (b), which correspond to the degeneracy types (A) and (B) (See Methods), show good agreement with the perturbation theory. However, the approximate eigenvectors in Figs (c) and (d), which are of the degeneracy type (C), are not in good quantitative agreement with the true vectors (although they exhibit qualitatively similar patterns). Thus, the degeneracy of the eigenvectors affects the performance of the perturbation approximation.

Indeed, quantitative node-wise comparison of the true and approximate vectors yields considerable discrepancy. The fact that the essential localization property of the vectors is qualitatively reproduced in Fig. 3 suggests that the true and approximate eigenvectors share similar characteristics when they are averaged over degenerate nodes and eigenvectors. In order to evaluate the performance of approximation while excluding the effect of degeneracy, we construct reduced degree-wise vectors from the true node-wise Laplacian eigenvectors, where vector components of the original node-wise Laplacian vectors are averaged over degenerate nodes having the same degree and over the degenerate eigenmodes having the same zero-th order eigenvalues as follows:

6 $${|{v}_{k}^{(\beta )}|}^{2}=\frac{1}{{N}_{k}{N}_{\beta }}\sum _{\{i:{k}_{i}=k\}}\sum _{\{\alpha :{{\rm{\Lambda }}}_{0}^{(\alpha )}=-{k}_{\beta }\}}{|{\varphi }_{i}^{(\alpha )}|}^{2}\mathrm{.}$$

Here, the degree index *k* runs from the minimum degree to the maximum degree of the network nodes, the reduced eigenvalue index *β* runs from the minimum to maximum of the zero-th order eigenvalues, ${N}_{k}$ is the number of degenerate nodes with degree *k*, and ${N}_{\beta }$ is the number of degenerate eigenmodes with the zero-th order eigenvalue $-{k}_{\beta }$, respectively. We then calculate the correlation coefficient *σ* between the true and approximate eigenvectors, defined by

7 $$\sigma (\beta )=\sqrt{\sum _{k}{|{v}_{k}^{(\beta )}|}^{2}{|{\tilde{v}}_{k}^{(\beta )}|}^{2}},$$

where ${\tilde{v}}_{k}^{(\beta )}$ is the reduced degree-wise vector obtained from the approximate eigenvectors. For comparison, we also generate *N* independent random eigenvectors whose components are randomly drawn from a uniform distribution over [−1, 1], normalize the vectors so that their norms become equal to 1, and calculated their correlations similarly. We exclude the uniform eigenvector ${\overrightarrow{\varphi }}^{(N)}=(\mathrm{1,}\,1,\,\cdots ,1)/\sqrt{N}$ from the analysis, which is exceptional and cannot be predicted by the perturbation theory.

Figure 4(e–g) show the correlation coefficient *σ* between the true and approximate eigenvectors with respect to the reduced eigenvalue index *β*. As can be seen in the figures, the correlation coefficient *σ* takes large values for non-degenerate eigenmodes, which are much larger than the correlation coefficient for random vectors and thus indicate similarity between the true and approximate vectors. It can be seen that non-degenerate vectors show larger correlations than degenerate vectors. It can be also observed that *σ* is higher near hubs for the BA, and near hubs and peripheries for the ER and CE. For other eigenvectors, *σ* can be as small as those of random vectors, indicating that the perturbation theory does not predict some of the eigenvectors well.

We stress that, although the correlation coefficients can be small for some of the eigenvectors, that is, the perturbation approximation does not predict them *quantitatively*, essential *qualitative* properties of the true eigenvectors such as the localizing nodes and the degree of localization are still reproduced well. This is because such properties are mainly determined by the degree of the nodes in the same degenerate block, which share statistically similar connectivities to the rest of the network. See Methods for the discussion on the accuracy of the perturbation approximation.

Thus, the perturbation theory can account for the eigenvector localization and degree-eigenvalue correspondence reasonably well. It reveals how degree heterogeneity and degeneracy lead to the eigenvector localization and the degree-eigenvalue correspondence. Furthermore, it clarifies why the representation of the Laplacian eigenvectors in Fig. 1, with respect to the eigenvector index *α* and the node index *i* both sorted in decreasing order of the eigenvalues and degrees, yields the clearly visible localized structures.

Our perturbation analysis also explains how similar the degrees of the nodes should be in order that the Laplacian eigenvector localizes on these nodes. From Eqs (9–11), we observe that the difference in the zero-th order eigenvalues in the denominator, ${{\rm{\Lambda }}}_{0}^{(\alpha )}-{{\rm{\Lambda }}}_{0}^{(\beta )}$, which is equal to the difference in the node degrees from Eq. (5), should be sufficiently smaller than the maximal range of the eigenvalues, ${{\rm{\Lambda }}}_{0}^{(N)}-{{\rm{\Lambda }}}_{0}^{\mathrm{(1)}}$, which is equal to ${k}_{{\rm{\max }}}-{k}_{{\rm{\min }}}$, in order to give a dominant contribution to the first-order correction to the eigenvector. (If this is not the case, all nodes in the network will give similar contributions to the perturbation correction and localization will not be observed). Thus, the degrees of the nodes should be similar in the sense that their difference is much smaller than the range of the entire degree distribution, ${k}_{1}-{k}_{N}$, as we mentioned in the introduction.

### Weighted and directed networks

Although we have so far presented the results only for non-directed and non-weighted networks, our analysis can straightforwardly be extended to directed and weighted networks. For directed networks, the adjacency matrix **A** is generally asymmetric. The weight of the edge from node *j* to node *i* is specified by the element *W*_*ij*_ of the weight matrix **W**. The Laplacian matrix of such a network is defined as

8 $${L}_{ij}={W}_{ij}{A}_{ij}-{k}_{i}^{{\rm{out}}}{\delta }_{i,j},$$

where ${k}_{i}^{{\rm{out}}}={\sum }_{j=1}^{N}{W}_{ji}{A}_{ji}$ is the outgoing degree of the *i*th node. The diagonal elements of this Laplacian matrix is of the order ${\mathcal{O}}(\langle {k}^{{\rm{out}}}\rangle )$, while non-diagonal elements are ${\mathcal{O}}({W}_{ij})$. Thus, if the network is sufficiently dense, $\langle {k}^{{\rm{out}}}\rangle \gg {W}_{ij}$ holds generally.

By introducing an expansion parameter $\varepsilon ^{\prime} =\langle W\rangle /\langle {k}^{{\rm{out}}}\rangle$, we can rewrite the Laplacian matrix as

9 $${\bf{L}}={{\bf{L}}}_{0}+\varepsilon ^{\prime} {{\bf{L}}}_{1},$$

whose elements

10 $${L}_{\mathrm{0,}ij}=-{k}_{i}^{{\rm{out}}}{\delta }_{i,j}\quad {\rm{and}}\quad {L}_{\mathrm{1,}ij}=\frac{\langle {k}^{{\rm{out}}}\rangle }{\langle W\rangle }{W}_{ij}{A}_{ij}$$

are of the same order, ${\mathcal{O}}(\langle {k}^{{\rm{out}}}\rangle )$. Thus, one can follow the perturbation analysis as described above with the generalized Laplacian matrix **L**.

As an illustrative example, Fig. 5 compares the true Laplacan eigenvectors and the result of the perturbation approximation of the real asymmetric neural network of *C. elegans*, which we used in Figs 1–4 after symmetrization. Note that the Laplacian matrix is now asymmetric and the elements of its eigenvectors can take complex values. We focus only on the localization pattern of the Laplacian eigenvectors and plot the absolute value of each vector component. As can be seen in the figure, the approximate eigenvectors can reproduce the localization pattern of the true eigenvectors qualitatively well.

### Regular lattices

Our argument suggests that the block-diagonal components representing the eigenvector localization can also be more or less observed even when the network is not *random*, but is formed by nodes with non-identical degrees. In order to verify this statement, we here consider a regular lattice network as shown in Fig. 6(a), which is composed of three types of nodes with different degrees. Specifically, one third of the nodes have degree *k* = 6, another one third have degree *k* = 4, and the rest have degree *k* = 2.

The zeroth-order unperturbed result is shown in Fig. 6(b). All eigenvectors degenerate into three classes at this stage, corresponding to the characteristic degrees *k* = 6, *k* = 4, and *k* = 2. Higher-order perturbations solve this degeneracy and mix the eigenvectors in each subset into three blocks, corresponding to *k* = 6, *k* = 4, and *k* = 2. Thus, at the first-order perturbation, the Laplacian eigenvectors show block-diagonal structures in the contour plot as shown in Fig. 6(c).

The above prediction is in good agreement with the true Laplacian eigenvectors obtained by direct numerical calculation, shown in Fig. 6(d). Thus, the degree heterogeneity generally leads to localized eigenvectors even in regular lattice networks. This result also suggests that the degree heterogeneity is the origin of the localization property of the Laplacian eigenvectors.

## Discussion

The Laplacian eigenvectors of networks with degree heterogeneity generally exhibit localization on the subset of nodes with close degrees and the Laplacian eigenvalues show clear degree-eigenvalue correspondence. We have proposed a simple explanation for the localization property of Laplacian eigenvectors using the degenerate perturbation theory. It clarifies how degree heterogeneity and degeneracy lead to the eigenvector localization and degree-eigenvalue correspondence. We analyzed three kinds of random networks with different statistical properties, and confirmed that our approach can reasonably account for the true Laplacian eigenvectors. We have also shown that the analysis can straightforwardly be extended to directed and weighted networks.

Our results show that the node degrees in heterogeneous networks correspond to the wavenumbers in regular lattices. Therefore, the degree of the node can play an essential role as the “natural coordinate” in describing the dynamics or patterns on networks with heterogeneous degree distributions, because they determine the eigenvalue and the subset of nodes that participate in the eigenvector. We conjecture that this partly accounts for why various network dynamics, plotted with respect to node degrees, often exhibit ordered patterns and provide us with physical interpretations.

In this study, we used the *C. elegans* neuronal network to illustrate the generality of the eigenvector localization and did not analyze its particular functional structures. Though extraction of functional structures from the *C. elegans* neuronal network is beyond the scope of the present study, we can observe a possible sign of such structures from the eigenvalues of the *C. elegans* network shown in Fig. 2(f); that is, there exists a small cluster of eigenvalues (3 ≤ *α* ≤ 12) separated from other eigenvalues, and correspondingly a tiny block structure exists on top of the diagonal structure in the Laplacian eigenvectors in Fig. 1(d). Such detailed structures of the Laplacian eigenvectors could reflect some functional structure of the neuronal network of *C. elegans*. In analyzing such detailed structures, broad degree heterogeneity, which yields the diagonal localized structure, might be disturbing, and spectral clustering methods based on the normalized Laplacian matrix^28^, which removes the effect of degree heterogeneity, could provide more useful information of the network.

Finally, we note that the localization property of eigenvectors is not restricted to network Laplacian matrices. We have recently formulated the advection equation for random networks^29^ and found that the eigenvectors of the advection matrix are also localized on a subset of nodes. This localization can also be accounted for by a similar perturbation analysis of the network, and the homogenization process on the network due to advection can be clearly visualized when plotted with respect to the node degrees. Further investigation of eigenvector localization on networks will provide us with insights into complex dynamics on networks.

## Methods

### Degenerate perturbation theory

Following a standard argument from quantum mechanics, we classify each eigenvector into the following three types according to its degeneracy: (A) non-degenerate, (B) degeneration that is solved at the first order, and (C) otherwise. We compute the approximate eigenvectors and eigenvalues by the first- and second-order perturbation theory, respectively. For each case, the perturbation corrections are given as follows (see Supplementary Information for the derivation).

For type (A), the first order correction is

11 $${|\alpha \rangle }_{1}=\sum _{\beta \ne \alpha }\frac{{}_{0}\langle \beta |{L}_{1}|\alpha \rangle _{0}}{{{\rm{\Lambda }}}_{0}^{(\alpha )}-{{\rm{\Lambda }}}_{0}^{(\beta )}}{|\beta \rangle }_{0}\mathrm{.}$$

The first order correction to the eigenvalue vanishes, i.e., ${{\rm{\Lambda }}}_{1}^{(\alpha )}={}_{0}\langle \alpha |{L}_{1}{|\alpha \rangle }_{0}=0$, because the diagonal elements of *L*_1_ are zero. The second order correction to the eigenvalue is given by ${{\rm{\Lambda }}}_{2}^{(\alpha )}={}_{0}\langle \alpha |{L}_{1}{|\alpha \rangle }_{1}$.

For type (B), we denote the degenerate eigenvectors corresponding to the same eigenvalue ${{\rm{\Lambda }}}_{0}^{(\alpha )}$ at the zeroth order as ${\alpha }_{1},\cdots ,{\alpha }_{m}$. We introduce new zeroth-order eigenvectors so that the degeneration is solved at the first-order perturbation as ${|{\tilde{\alpha }}_{i}\rangle }_{0}={\sum }_{j=1}^{m}{b}_{i,j}{|{\alpha }_{j}\rangle }_{0},$ where the mixing coefficients ${b}_{i,j}$ are the eigenvectors of the matrix **V** defined by ${V}_{ij}={}_{0}|{\alpha }_{i}\rangle {L}_{1}{|{\alpha }_{j}\rangle }_{0}$ ($i,j=\mathrm{1,}\,\cdots ,m$). Namely, they satisfy the secular eigenvalue equation ${\sum }_{j=1}^{m}{V}_{kj}{b}_{i,j}={{\rm{\Lambda }}}_{1}^{({\alpha }_{i})}{b}_{i,k},$ where ${{\rm{\Lambda }}}_{1}^{({\alpha }_{i})}$ gives the first-order correction to the Laplacian eigenvalue. The first-order correction to the eigenvector is

12 $${|{\tilde{\alpha }}_{i}\rangle }_{1}=\sum _{\beta \ne \alpha }\frac{{}_{0}\langle \beta |{L}_{1}{|{\tilde{\alpha }}_{i}\rangle }_{0}}{{{\rm{\Lambda }}}_{0}^{(\alpha )}-{{\rm{\Lambda }}}_{0}^{(\beta )}}[\sum _{j=1}^{m}\,\frac{{}_{0}\langle {\tilde{\alpha }}_{j}|{L}_{1}{|\beta \rangle }_{0}}{{{\rm{\Lambda }}}_{1}^{({\alpha }_{i})}-{{\rm{\Lambda }}}_{1}^{({\alpha }_{j})}}{|{\tilde{\alpha }}_{j}\rangle }_{0}+{|\beta \rangle }_{0}],$$

where the summation symbol with $\beta \ne \alpha$ indicates that the index *β* runs over all eigenvectors except for the degenerate ones, i.e., ${\alpha }_{1},\cdots ,{\alpha }_{m}$. The second order correction to the eigenvalue is given by ${{\rm{\Lambda }}}_{2}^{({\alpha }_{i})}={}_{0}\langle {\tilde{\alpha }}_{i}|{L}_{1}{|{\tilde{\alpha }}_{i}\rangle }_{1}$.

For type (C), suppose that a subset of the eigenvalues ${\tilde{\alpha }}_{1},\cdots ,{\tilde{\alpha }}_{n}$ ($n\le m$) is still degenerate at the first order perturbation. In this case, the zeroth-order eigenvector is further redefined as ${|{\tilde{\tilde{\alpha }}}_{i}\rangle }_{0}={\sum }_{j\mathrm{=1}}^{n}{c}_{i,j}{|{\tilde{\alpha }}_{j}\rangle }_{0},$ where ${c}_{i,j}$ are given by the eigenvectors of the matrix **W** defined as ${W}_{kj}={\sum }_{\beta \ne \alpha }({}_{0}|{\tilde{\alpha }}_{k}\rangle {L}_{1}{|\beta \rangle }_{0}{}_{0}\langle \beta |{L}_{1}{|{\tilde{\alpha }}_{j}\rangle }_{0})/({{\rm{\Lambda }}}_{0}^{(\alpha )}-{{\rm{\Lambda }}}_{0}^{(\beta )})$. The first-order correction to the eigenvector is given by

13 $${|{\tilde{\tilde{\alpha }}}_{i}\rangle }_{1}=\sum _{\beta \ne \alpha }\frac{{}_{0}\langle \beta |{L}_{1}{|{\tilde{\tilde{\alpha }}}_{i}\rangle }_{0}}{{{\rm{\Lambda }}}_{0}^{(\alpha )}-{{\rm{\Lambda }}}_{0}^{(\beta )}}[\sum _{k=n+1}^{m}\frac{{}_{0}\langle {\tilde{\alpha }}_{k}|{L}_{1}{|\beta \rangle }_{0}}{{{\rm{\Lambda }}}_{1}^{({\alpha }_{i})}-{{\rm{\Lambda }}}_{1}^{({\alpha }_{k})}}{|{\tilde{\alpha }}_{k}\rangle }_{0}+{|\beta \rangle }_{0}]\mathrm{.}$$

The first order correction ${{\rm{\Lambda }}}_{1}^{({\alpha }_{i})}$ to the eigenvalue is given as in (B), and the second order correction ${{\rm{\Lambda }}}_{2}^{({\alpha }_{i})}$ is determined from ${\sum }_{j\mathrm{=1}}^{m}{W}_{kj}{c}_{i,j}={{\rm{\Lambda }}}_{2}^{({\alpha }_{i})}{c}_{i,k}$.

If some eigenvectors are still degenerate even at the second-order perturbation, one can further introduce new zeroth-order eigenvectors so that the degeneracy is solved at the higher-order perturbation. However, for simplicity, we do not consider the higher-order perturbations. Therefore, the eigenvectors are not completely determined in this case.

### Accuracy of the perturbation approximation

As is well known, it is generally difficult to prove the convergence of the perturbation series. Accuracy of the perturbation approximation may roughly be assessed by looking at the ratio ${|}_{0}\langle \beta |{L}_{1}{|\alpha \rangle }_{0}|/|{{\rm{\Lambda }}}_{0}^{(\alpha )}-{{\rm{\Lambda }}}_{0}^{(\beta )}|$ for each pair of non-degenerate eigenmodes $\alpha$ and $\beta$. If this ratio is sufficiently small, the contribution of the zeroth-order eigenvector $|\beta {\rangle }_{0}$ to the first-order correction $|\alpha {\rangle }_{1}$ will be accurately evaluated.

In the present case, $|{}_{0}\langle \beta |{L}_{1}{|\alpha \rangle }_{0}|={A}_{\beta \alpha }$ is the element of the adjacency matrix and takes either 1 or 0, while $|{{\rm{\Lambda }}}_{0}^{(\alpha )}-{{\rm{\Lambda }}}_{0}^{(\beta )}|=|{k}_{\alpha }-{k}_{\beta }|$ is the difference between the characteristic degrees of the corresponding eigenvectors and is greater than 1 (See Methods). Therefore, if ${A}_{\beta \alpha }\mathrm{=1}$ and *k*_*β*_ is close to *k*_*α*_, the above ratio may not be small, namely, the contribution from $|\beta {\rangle }_{0}$ to the first-order correction $|\alpha {\rangle }_{1}$ may be inaccurate at the nodes with degree *k*_*β*_.

We note, however, that the perturbation theory can still qualitatively account for the localization property in such cases. In Fig. 1, the eigenvector $|\alpha \rangle$ is localized at the diagonal nodes whose indices satisfy $i\simeq \alpha$ and whose degrees are close to *k*_*α*_, because the node indices {*i*} are sorted so that ${k}_{1}\ge {k}_{2}\ge \cdots \ge {k}_{N}$. Our main aim is to explain that $|\alpha \rangle$ is almost vanishing at the non-diagonal nodes with $|i-\alpha |\gg 1$. The degree *k*_*β*_ of such non-diagonal nodes are generally far from *k*_*α*_ for networks with degree heterogeneity, so that the above ratio would generally be small and the first-order correction ${|\alpha \rangle }_{1}$ would reliably be obtained for such nodes. Thus, the perturbation theory can account for why the eigenvector takes tiny components at non-diagonal nodes even if they can give inaccurate results for diagonal nodes.

Indeed, as explained in the Results, the theory can reproduce the true eigenvalues very accurately and account for the localization property qualitatively well for all three networks shown in Fig. 1. Furthermore, it can even predict precise localizing patterns quantitatively well for some of the eigenvectors.

## Electronic supplementary material

Supplementary Information
