# Erratum: Localization of Laplacian eigenvectors on random networks

**DOI:** 10.1038/s41598-017-06298-6

**Published:** 2017-09-06

**Authors:** Shigefumi Hata, Hiroya Nakao

**Affiliations:** 1 Department of Physics and Astronomy, Kagoshima University, Kagoshima, 890-0065 Japan; 2 Department of Systems and Control Engineering, Tokyo Institute of Technology, Tokyo, 152-8552 Japan

*Scientific Reports* **7**:1121; doi:10.1038/s41598-017-01010-0; Article published online 25 April 2017

This Article contains errors in the Methods section under the sub-heading “Degenerate perturbation theory”, where:

$${V}_{ij}={}_{0}|{\alpha }_{i}\rangle {L}_{1}{|{\alpha }_{j}\rangle}_{0}$$

should read:

$${V}_{ij}={}_{0}\langle {\alpha }_{i}|{L}_{1}{|{\alpha }_{j}\rangle}_{0}$$

In addition,

$${W}_{kj}={{\rm{\Sigma }}}_{\beta \ne \alpha }({}_{0}|{\tilde{\alpha }}_{k}\rangle {L}_{1}{|\beta \rangle }_{0}\,{}_{0}\langle \beta |{L}_{1}{|{\tilde{\alpha }}_{j}\rangle }_{0})/({{{\rm{\Lambda }}}_{0}}^{(\alpha )}-{{{\rm{\Lambda }}}_{0}}^{(\beta )})$$

should read:

$${W}_{kj}={{\rm{\Sigma }}}_{\beta \ne \alpha }({}_{0}\langle {\tilde{\alpha }}_{k}|{L}_{1}{|\beta \rangle }_{0}\,{}_{0}\langle \beta |{L}_{1}{|{\tilde{\alpha }}_{j}\rangle }_{0})/({{{\rm{\Lambda }}}_{0}}^{(\alpha )}-{{{\rm{\Lambda }}}_{0}}^{(\beta )})$$

