# Supplementary material for: Localization of Laplacian eigenvectors on random networks
Source: Sci Rep. 2017 Apr 25;7:1121. doi: 10.1038/s41598-017-01010-0 (PMC5430689; doi:10.1038/s41598-017-01010-0)
Supplement: Supplementary file 1 — Supplementary Information [file 41598_2017_1010_MOESM1_ESM.pdf]

Localization of Laplacian eigenvectors on random networks
# - Supplementary Information -

Shigefumi Hata[1] and Hiroya Nakao[2]

[1] *Department of Physics and Astronomy, Kagoshima University, Kagoshima, 890-0065, Japan*

[2] *Department of Systems and Control Engineering, Tokyo Institute of Technology, Tokyo 152-8552, Japan*

In this Supplementary Information, complete derivation of the perturbation corrections, Eqs. (9)-(11), is provided. As remarked in the main text, the Laplacian matrix $\mathbf{L}$ can be splitted as

$$\mathbf{L} = \mathbf{L}_0 + \epsilon \mathbf{L}_1, \tag{S1}$$

where $\epsilon = \langle k \rangle^{-1}$, so that the elements

$$L_{0,ij} = -k_i \delta_{i,j} \quad \text{and} \quad L_{1,ij} = \langle k \rangle A_{ij} \tag{S2}$$

of the linear operators $\mathbf{L}_0$ and $\mathbf{L}_1$ are of the same order, $\mathcal{O}(\langle k \rangle)$. In sufficiently dense networks, i.e., $\langle k \rangle \gg 1$, the order parameter ϵ is small. We thus apply the standard perturbation theory from quantum mechanics [1, 2] to approximate the Laplacian eigenvectors using ϵ as the expansion parameter.

For convenience, the bra-ket notation is hereafter employed. We denote the Laplacian eigenvector of the α th mode as $\vec{\phi}^{(\alpha)} = |\alpha\rangle$ and drop the summation symbol as $\sum_{j=1}^{N} L_{ij} \phi_j^{(\alpha)} = L\,|\alpha\rangle$. We expand the Laplacian eigenvectors $|\alpha\rangle$ and eigenvalues $\Lambda^{(\alpha)}$ in series of the order parameter ϵ, that is,

$$|\alpha\rangle = |\alpha\rangle_0 + \epsilon\,|\alpha\rangle_1 + \epsilon^2\,|\alpha\rangle_2 + \cdots, \tag{S3}$$

$$\Lambda^{(\alpha)} = \Lambda_0^{(\alpha)} + \epsilon \Lambda_1^{(\alpha)} + \epsilon^2 \Lambda_2^{(\alpha)} + \cdots. \tag{S4}$$

As usual, we assume that the unperturbed eigenvectors are orthonormalized, i.e, ${}_0\langle \beta|\alpha\rangle_0 = \delta_{\alpha,\beta}$, and that the higher-order corrections are orthogonal to the unperturbed eigenvector, i.e., $\langle \alpha|\alpha\rangle_j = 0$ for $j = 1, 2, \cdots$. Substituting these expressions into the eigenvalue equation, $L\,|\alpha\rangle = \Lambda^{(\alpha)}\,|\alpha\rangle$, the

following set of equations is obtained:

$$\left(L_0 - \Lambda_0^{(\alpha)}\right) |\alpha\rangle_0 = 0, \tag{S5}$$

$$\left(L_0 - \Lambda_0^{(\alpha)}\right) |\alpha\rangle_1 = -\left(L_1 - \Lambda_1^{(\alpha)}\right) |\alpha\rangle_0\,, \tag{S6}$$

$$\left(L_0 - \Lambda_0^{(\alpha)}\right) |\alpha\rangle_2 = -\left(L_1 - \Lambda_1^{(\alpha)}\right) |\alpha\rangle_1 + \Lambda_2^{(\alpha)} |\alpha\rangle_0\,. \tag{S7}$$

A. Eigenvectors of the unperturbed system

We first calculate the eigenvectors and eigenvalues of the unperturbed system. Examining Eq. (S5), we can find the unperturbed eigenvectors $|\alpha\rangle_0$ and eigenvalues $\Lambda_0^{(\alpha)}$ exactly as

$$|\alpha\rangle_0 = (0, \cdots, 0, \overset{\overset{\alpha}{\vee}}{1}, 0, \cdots, 0), \tag{S8}$$

$$\Lambda_0^{(\alpha)} = -k_\alpha, \tag{S9}$$

for $\alpha = 1, 2, \cdots, N$. Each eigenvector is a unit vector with a single non-vanishing element 1 at the network node α. The corresponding eigenvalue is the negative of the node degree. Note that, in networks, there are generally multiple nodes having the same degree. This indicates that the corresponding eigenvectors are degenerate, namely, they belong to the same eigenvalues in the absence of the perturbation.

B. First-order approximation

Here we derive the first-order perturbative corrections to the Laplacian eigenvectors. As noted above, degenerate eigenvectors may exist in the unperturbed system. Thus, we need to employ the degenerate perturbation theory for calculating the corrections to the unperturbed eigenvectors. Each eigenvector is classified into the following three types according to its degeneracy: (A) non-degenerate, (B) degeneration is solved at the first order, and (C) otherwise. For each case, we calculate the first-order corrections to the eigenvectors and second-order corrections to the eigenvalues.

(A) Non-degenerate case

Multiplying Eq. (S6) by the bra ${}_0\langle\alpha|$ from the left yields

$$\Lambda_1^{(\alpha)} = {}_0\langle\alpha|L_1|\alpha\rangle_0 = \langle k\rangle A_{\alpha\alpha} = 0. \tag{S10}$$

Thus, the first-order correction to the Laplacian eigenvalue vanishes.

To obtain the first-order correction $|\alpha\rangle_1$ to the α-th vector, we expand it over the set of eigenvectors $|\beta\rangle_0$ ($\beta \neq \alpha$) as

$$|\alpha\rangle_1 = \sum_{\beta \neq \alpha} {}_0\langle\beta|\alpha\rangle_1 \, |\beta\rangle_0 \,, \tag{S11}$$

where the eigenvector $|\alpha\rangle_0$ is excluded from the expansion. Multiplying Eq. (S6) by a bra ${}_0\langle\beta|$ ($\beta \neq \alpha$) corresponding to a different eigenvalue $\Lambda_0^{(\beta)}$ from the left, we obtain the expansion coefficients in Eq. (S11) as

$${}_0\langle\beta|\alpha\rangle_1 = \frac{{}_0\langle\beta|L_1|\alpha\rangle_0}{\Lambda_0^{(\alpha)} - \Lambda_0^{(\beta)}}. \tag{S12}$$

Thus, the first-order correction to the eigenvector is calculated as

$$|\alpha\rangle_1 = \sum_{\beta \neq \alpha} \frac{{}_0\langle\beta|L_1|\alpha\rangle_0}{\Lambda_0^{(\alpha)} - \Lambda_0^{(\beta)}} \, |\beta\rangle_0 \,. \tag{S13}$$

We calculate the eigenvalues to the second order. Multiplying Eq. (S7) by the bra ${}_0\langle\alpha|$ from the left gives

$$\Lambda_2^{(\alpha)} = {}_0\langle\alpha|L_1|\alpha\rangle_1 \,. \tag{S14}$$

Thus, by substituting expression (S13) to Eq. (S14), we can calculate the second-order correction to the eigenvalue as

$$\Lambda_2^{(\alpha)} = \sum_{\beta \neq \alpha} \frac{\left({}_0\langle\beta|L_1|\alpha\rangle_0\right)^2}{\Lambda_0^{(\alpha)} - \Lambda_0^{(\beta)}}. \tag{S15}$$

(B) Degeneration is solved at the first order

Suppose that the unperturbed eigenvectors $\{\ |\alpha_1\rangle_0 \,, |\alpha_2\rangle_0 \,, \cdots |\alpha_g\rangle_0\ \}$ belong to the same eigenvalue $\Lambda_0^{(\alpha)}$. In this case, linearly transformed vectors

$$|\tilde{\alpha}_i\rangle_0 = \sum_{j=1}^{g} b_{i,j} \, |\alpha_j\rangle_0 \quad (i = 1, ..., g), \tag{S16}$$

are also solutions of Eq. (S5) and form another orthonormal basis. We determine the transformation coefficients $\{b_{i,j}\}$ so that the degeneration is solved at the first-order correction.

Expanding the eigenvectors of the original system over the transformed eigenvectors as

$$|\tilde{\alpha}_i\rangle = |\tilde{\alpha}_i\rangle_0 + \epsilon \, |\tilde{\alpha}_i\rangle_1 + \epsilon^2 \, |\tilde{\alpha}_i\rangle_2 + \cdots \,, \tag{S17}$$

we obtain the first-order perturbation equation for the degenerate eigenvectors as

$$\left(L_0 - \Lambda_0^{(\alpha_i)}\right) |\tilde{\alpha}_i\rangle_1 = -\left(L_1 - \Lambda_1^{(\alpha_i)}\right) |\tilde{\alpha}_i\rangle_0 \,, \tag{S18}$$

where $\Lambda_1^{(\alpha_i)}$ depends on i. Substituting (S16) into Eq. (S18), we obtain

$$\left(L_0 - \Lambda_0^{(\alpha_i)}\right) |\tilde{\alpha}_i\rangle_1 = -\sum_{j=1}^{g} \left(L_1 - \Lambda_1^{(\alpha_i)}\right) b_{i,j} \, |\alpha_j\rangle_0 \,. \tag{S19}$$

By multiplying the bra of the degenerate vector ${}_0\langle\alpha_k|$ $(k = 1, \cdots, g)$ from the left, we obtain

$$0 = -\sum_{j=1}^{g} b_{i,j} \, {}_0\langle\alpha_k|L_1|\alpha_j\rangle_0 + \sum_{j=1}^{g} b_{i,j}\Lambda_1^{(\alpha_i)} \, {}_0\langle\alpha_k|\alpha_j\rangle_0 \,, \tag{S20}$$

which yields

$$\sum_{j=1}^{g} V_{kj} b_{i,j} = \Lambda_1^{(\alpha_i)} b_{i,k} \tag{S21}$$

where

$$V_{kj} = {}_0\langle\alpha_k|L_1|\alpha_j\rangle_0 \,. \tag{S22}$$

Thus, by solving the characteristic (secular) equation (S21), the transformation coefficients $\{b_{i,j}\}$ in Eq. (S16) can be obtained as the elements of the eigenvector of this matrix. The corresponding eigenvalues $\Lambda_1^{(\alpha_i)}$ $(i = 1, ..., g)$ give the first-order correction to the Laplacian eigenvalues.

Next, we expand the first-order correction $|\tilde{\alpha}_i\rangle_1$ over the set of unperturbed kets as

$$|\tilde{\alpha}_i\rangle_1 = {\sum_{j=1}^{g}}' \, {}_0\langle\tilde{\alpha}_j|\tilde{\alpha}_i\rangle_1 \, |\tilde{\alpha}_j\rangle_0 + \sum_{\beta\neq\alpha} {}_0\langle\beta|\tilde{\alpha}_i\rangle_1 \, |\beta\rangle_0 \,, \tag{S23}$$

where the summation symbol ${\sum_{j=1}^{g}}'$ with a prime means that the index j runs from 1 to g while skipping i. By multiplying ${}_0\langle\tilde{\alpha}_j| \, L_1$ $(i \neq j)$ from the left, we obtain

$$\Lambda_1^{(\alpha_i)} \, {}_0\langle\tilde{\alpha}_j|\tilde{\alpha}_i\rangle_1 = \Lambda_1^{(\alpha_j)} \, {}_0\langle\tilde{\alpha}_j|\tilde{\alpha}_i\rangle_1 + \sum_{\beta\neq\alpha} {}_0\langle\beta|\tilde{\alpha}_i\rangle_1 \, {}_0\langle\tilde{\alpha}_j|L_1|\beta\rangle_0 \,, \tag{S24}$$

which yields

$${}_0\langle\tilde{\alpha}_j|\tilde{\alpha}_i\rangle_1 = \sum_{\beta\neq\alpha} \frac{{}_0\langle\tilde{\alpha}_j|L_1|\beta\rangle_0}{\Lambda_1^{(\alpha_i)} - \Lambda_1^{(\alpha_j)}} \, {}_0\langle\beta|\tilde{\alpha}_i\rangle_1 \,. \tag{S25}$$

Thus, the expansion (S23) is rewritten as

$$|\tilde{\alpha}_i\rangle_1 = \sum_{\beta\neq\alpha} {}_0\langle\beta|\tilde{\alpha}_i\rangle_1 \left[{\sum_{j=1}^{g}}' \frac{{}_0\langle\tilde{\alpha}_j|L_1|\beta\rangle_0}{\Lambda_1^{(\alpha_i)} - \Lambda_1^{(\alpha_j)}} \, |\tilde{\alpha}_j\rangle_0 + |\beta\rangle_0 \right] . \tag{S26}$$

By multiplying Eq. (S18) by a bra ${}_0\langle\beta|$ corresponding to a different eigenvalue $\Lambda_0^{(\beta)}$ from the left, we obtain

$$\left(\Lambda_0^{(\beta)} - \Lambda_0^{(\alpha)}\right) {}_0\langle\beta|\tilde{\alpha}_i\rangle_1 = {}_0\langle\beta|L_1|\tilde{\alpha}_i\rangle\,, \tag{S27}$$

namely,

$$ {}_0\langle\beta|\tilde{\alpha}_i\rangle_1 = \frac{{}_0\langle\beta|L_1|\tilde{\alpha}_i\rangle_0}{\Lambda_0^{(\alpha)} - \Lambda_0^{(\beta)}}. \tag{S28}$$

Thus, the first-order correction to the degenerate eigenvectors can be calculated as

$$|\tilde{\alpha}_i\rangle_1 = \sum_{\beta\neq\alpha} \frac{{}_0\langle\beta|L_1|\tilde{\alpha}_i\rangle_0}{\Lambda_0^{(\alpha)} - \Lambda_0^{(\beta)}} \left[\sum_{j=1}^{g}{}' \frac{{}_0\langle\tilde{\alpha}_j|L_1|\beta\rangle_0}{\Lambda_1^{(\alpha_i)} - \Lambda_1^{(\alpha_j)}} |\tilde{\alpha}_j\rangle_0 + |\beta\rangle_0 \right]. \tag{S29}$$

As before, we calculate the eigenvalues to the second order. Multiplying the second-order equation

$$\left(L_0 - \Lambda_0^{(\alpha_i)}\right)|\tilde{\alpha}_i\rangle_2 = -\left(L_1 - \Lambda_1^{(\alpha_i)}\right)|\tilde{\alpha}_i\rangle_1 + \Lambda_2^{(\alpha_i)}|\tilde{\alpha}_i\rangle_0\,, \tag{S30}$$

by the bra ${}_0\langle\tilde{\alpha}_i|$ from the left yields

$$\Lambda_2^{(\alpha_i)} = {}_0\langle\tilde{\alpha}_i|L_1|\tilde{\alpha}_i\rangle_1\,. \tag{S31}$$

We can substitute Eq. (S29) to Eq. (S31) to obtain the second-order correction to the eigenvalue as

$$\Lambda_2^{(\alpha_i)} = \sum_{\beta\neq\alpha} \frac{{}_0\langle\beta|L_1|\tilde{\alpha}_i\rangle_0}{\Lambda_0^{(\alpha)} - \Lambda_0^{(\beta)}} \left[\sum_{j=1}^{g}{}' \frac{\left({}_0\langle\tilde{\alpha}_j|L_1|\beta\rangle_0\right)^2}{\Lambda_1^{(\alpha_i)} - \Lambda_1^{(\alpha_j)}} + {}_0\langle\tilde{\alpha}_i|L_1|\beta\rangle_0 \right]. \tag{S32}$$

(C) Otherwise

If the degeneration is not solved at the first-order perturbation, eigenvectors $|\tilde{\alpha}_i\rangle_0$ in Eq. (S16) should be transformed again so that the degeneration is solved at the second-order perturbation. Suppose that eigenvectors $\{\ |\tilde{\alpha}_1\rangle_0\,,\ |\tilde{\alpha}_2\rangle_0\,,\ \cdots\,,\ |\tilde{\alpha}_h\rangle_0\ \}$ are still degenerate at the first order, that is, $\Lambda_1^{(\alpha_1)} = \Lambda_1^{(\alpha_2)} = \cdots = \Lambda_1^{(\alpha_h)}$, while the rest of eigenvectors $\{\ |\tilde{\alpha}_{h+1}\rangle_0\,, |\tilde{\alpha}_{h+2}\rangle_0\,, \cdots\,, |\tilde{\alpha}_g\rangle_0\ \}$ now belong to the different eigenvalues. We further transform the degenerate eigenvectors as

$$|\tilde{\tilde{\alpha}}_i\rangle_0 = \sum_{j=1}^{h} c_{i,j}\,|\tilde{\alpha}_j\rangle_0\,, \tag{S33}$$

and expand the α-th eigenvector. By multiplying the second-order equation

$$\left(L_0 - \Lambda_0^{(\alpha_i)}\right) |\tilde{\tilde{\alpha}}_i\rangle_2 = -\left(L_1 - \Lambda_1^{(\alpha_i)}\right) |\tilde{\tilde{\alpha}}_i\rangle_1 + \Lambda_2^{(\alpha_i)} |\tilde{\tilde{\alpha}}_i\rangle_0 \,, \tag{S34}$$

from the left by a bra ${}_0\langle\tilde{\alpha}_k|$ ($k = 1, \cdots, h$), we obtain

$$0 = -{}_0\langle\tilde{\alpha}_k|L_1|\tilde{\tilde{\alpha}}_i\rangle_1 + \Lambda_1^{(\alpha_i)}{}_0\langle\tilde{\alpha}_k|\tilde{\tilde{\alpha}}_i\rangle_1 + \Lambda_2^{(\alpha_i)}{}_0\langle\tilde{\alpha}_k|\tilde{\tilde{\alpha}}_i\rangle_0 \,. \tag{S35}$$

Next, by expanding the first-order correction $|\tilde{\tilde{\alpha}}_i\rangle_1$ over $\{|\tilde{\alpha}_j\rangle_0\}$ ($j = 1, \cdots, g$) and the other eigenvectors $\{|\beta\rangle_0\}$ ($\beta \neq \alpha$) which belong to the different eigenvalues from the $\tilde{\tilde{\alpha}}_i$-th mode under the first-order approximation;

$$|\tilde{\tilde{\alpha}}_i\rangle_1 = \sum_{j=1}^{g} {}_0\langle\tilde{\alpha}_j|\tilde{\tilde{\alpha}}_i\rangle_1 \, |\tilde{\alpha}_j\rangle_0 + \sum_{\beta\neq\alpha} {}_0\langle\beta|\tilde{\tilde{\alpha}}_i\rangle_1 \, |\beta\rangle_0 \,. \tag{S36}$$

Perturbation corrections to degenerate eigenvectors are generally not determined until higher-order perturbations that completely resolve the degeneration are taken into account [2]. In the present case, we examine the perturbation equations only up to the second order. Therefore, if some eigenvectors still remain degenerate at this order, perturbation corrections to these vectors cannot be determined from Eqs. (S5)-(S7). In order to avoid this problem, it is convenient to employ the *intermediate normalization* [2]

$${}_0\langle\tilde{\alpha}_j|\tilde{\tilde{\alpha}}_i\rangle = \delta_{i,j}, \tag{S37}$$

for $i = 1, \cdots, h$ and $j = h + 1, \cdots, g$, instead of the standard normalization $\langle\tilde{\alpha}_j|\tilde{\tilde{\alpha}}_i\rangle = \delta_{i,j}$. From Eq. (S37), one can easily check that

$${}_0\langle\tilde{\alpha}_j|\tilde{\tilde{\alpha}}_i\rangle_1 = 0, \tag{S38}$$

holds.

Thus, the eigenvectors $\{|\tilde{\alpha}_j\rangle_0\}$ ($j = h + 1, \cdots, g$) are not used for the expansion in Eq. (S36). By plugging the expansions (S33) and (S36) to Eq. (S35), we obtain

$$\Lambda_1^{(\alpha_k)}{}_0\langle\tilde{\alpha}_k|\tilde{\tilde{\alpha}}_i\rangle_1 + \sum_{\beta\neq\alpha} {}_0\langle\beta|\tilde{\tilde{\alpha}}_i\rangle_1 \, {}_0\langle\tilde{\alpha}_k|L_1|\beta\rangle_0 = \Lambda_1^{(\alpha_i)}{}_0\langle\tilde{\alpha}_k|\tilde{\tilde{\alpha}}_i\rangle_1 + \Lambda_2^{(\alpha)} c_{i,k}, \tag{S39}$$

for $i = 1, \cdots, h$ and $k = 1, \cdots, h$. By using the fact that $\Lambda_1^{(\alpha_i)} = \Lambda_1^{(\alpha_k)}$, we have

$$\begin{aligned} \Lambda_2^{(\alpha)} c_{i,k} &= \sum_{\beta\neq\alpha} {}_0\langle\beta|\tilde{\tilde{\alpha}}_i\rangle_1 \, {}_0\langle\tilde{\alpha}_k|L_1|\beta\rangle_0 \\ &= \sum_{j=1}^{h} c_{i,j} \left[\sum_{\beta\neq\alpha} \frac{{}_0\langle\tilde{\alpha}_k|L_1|\beta\rangle_0 \, {}_0\langle\beta|L_1|\tilde{\alpha}_j\rangle_0}{\Lambda_0^{(\alpha)} - \Lambda_0^{(\beta)}} \right]. \end{aligned} \tag{S40}$$

Thus, by solving the characteristic equation

$$\Lambda_2^{(\alpha)} c_{i,k} = \sum_{j=1}^{h} W_{kj} c_{i,j}, \tag{S41}$$

where

$$W_{kj} = \sum_{\beta \neq \alpha} \frac{{}_0\langle \tilde{\alpha}_k | L_1 | \beta \rangle_0 \, {}_0\langle \beta | L_1 | \tilde{\alpha}_j \rangle_0}{\Lambda_0^{(\alpha)} - \Lambda_0^{(\beta)}}, \tag{S42}$$

the transformation coefficients $\{c_{i,j}\}$ in Eq. (S33) are obtained as the elements of the eigenvector associated with the eigenvalue $\Lambda_2^{(\alpha)}$. This gives the second-order corrections to the Laplacian eigenvalues.

Now, by multiplying Eq. (S36) by ${}_0\langle \tilde{\alpha}_k | \, L_1$ $(k = h+1, \cdots, g)$ from the left, we obtain

$$\begin{aligned} \Lambda_1^{(\alpha_i)} \, {}_0\langle \tilde{\alpha}_k | \tilde{\tilde{\alpha}}_i \rangle_1 &= \sum_{j=h+1}^{g} {}_0\langle \tilde{\alpha}_j | \tilde{\tilde{\alpha}}_i \rangle_1 \, \Lambda_1^{(\alpha_j)} \, {}_0\langle \tilde{\alpha}_k | \tilde{\alpha}_j \rangle_0 + \sum_{\beta \neq \alpha} {}_0\langle \beta | \tilde{\tilde{\alpha}}_i \rangle_1 \, {}_0\langle \tilde{\alpha}_k | L_1 | \beta \rangle_0 \\ &= \Lambda_1^{(\alpha_k)} \, {}_0\langle \tilde{\alpha}_k | \tilde{\tilde{\alpha}}_i \rangle_1 + \sum_{\beta \neq \alpha} {}_0\langle \beta | \tilde{\tilde{\alpha}}_i \rangle_1 \, {}_0\langle \tilde{\alpha}_k | L_1 | \beta \rangle_0 \, . \end{aligned} \tag{S43}$$

Thus, we have

$${}_0\langle \tilde{\alpha}_k | \tilde{\tilde{\alpha}}_i \rangle_1 = \sum_{\beta \neq \alpha} \frac{{}_0\langle \beta | \tilde{\tilde{\alpha}}_i \rangle_1 \, {}_0\langle \tilde{\alpha}_k | L_1 | \beta \rangle_0}{\Lambda_1^{(\alpha_i)} - \Lambda_1^{(\alpha_k)}}. \tag{S44}$$

By multiplying the first-order equation from the left by ${}_0\langle \beta |$, we obtain

$$\left(\Lambda_0^{(\beta)} - \Lambda_0^{(\alpha)} \right) {}_0\langle \beta | \tilde{\tilde{\alpha}}_i \rangle_1 = - {}_0\langle \beta | L_1 | \tilde{\tilde{\alpha}}_i \rangle_0 \, , \tag{S45}$$

namely,

$${}_0\langle \beta | \tilde{\tilde{\alpha}}_i \rangle_1 = \frac{{}_0\langle \beta | L_1 | \tilde{\tilde{\alpha}}_i \rangle_0}{\Lambda_0^{(\alpha)} - \Lambda_0^{(\beta)}}. \tag{S46}$$

From Eqs. (S36), (S44) and (S46), the first-order correction $\left| \tilde{\tilde{\alpha}}_i \right\rangle_1$ is obtained as

$$|\tilde{\tilde{\alpha}}_i\rangle_1 = \sum_{\beta \neq \alpha} \frac{{}_0\langle \beta | L_1 | \tilde{\tilde{\alpha}}_i \rangle_0}{\Lambda_0^{(\alpha)} - \Lambda_0^{(\beta)}} \left[\sum_{k=h+1}^{g} \frac{{}_0\langle \tilde{\alpha}_k | L_1 | \beta \rangle_0}{\Lambda_1^{(\alpha_i)} - \Lambda_1^{(\alpha_k)}} |\tilde{\alpha}_k\rangle_0 + |\beta\rangle_0 \right]. \tag{S47}$$

Note that the *intermediate normalization* ${}_0\langle \tilde{\alpha}_j | \tilde{\tilde{\alpha}}_i \rangle = \delta_{i,j}$ which we employed in the calculation of the first-order correction to the eigenvectors, and the standard normalization $\langle \tilde{\alpha}_j | \tilde{\tilde{\alpha}}_i \rangle = \delta_{i,j}$ become equivalent only in the limit $\epsilon \to 0$. Thus, Eq. (S47) is only an approximation to the exact first-order correction. Therefore, in the present case, the approximate eigenvectors may not be in good agreement with the true eigenvectors as compared with those in the case (A) or (B).

---

[1] Sakurai J. J. & Napolitano J. J. Modern Quantum Mechanics (Pearson Education Ltd., London, 2013).

[2] Hirschfelder J. O. & Certain P. R. Degenerate RS perturbation theory. *J. Chem. Phys.* **60**, 1118 (1974).
